# Continuing Professional Development – Answers

**DOI:** 10.1002/jmrs.627

**Published:** 2022-12-01

Maximise your CPD by reading the selected article and answer the five questions. Please remember to self‐claim your CPD and retain your supporting evidence. Answers available online at www.asmirt.org/news-and-publications/jmrs.

## Answers to Questions Published in Previous Issues

### Volume 68, Issue 4 December 2021

Erskine B, Bradley P, Joseph T, Yeh S, Clements W. Comparing the accuracy and complications of peripherally inserted central catheter (PICC) placement using fluoroscopic and the blind pushing technique. *J Med Radiat Sci*. 2021 Dec; 68(4): 349–355. https://doi.org/10.1002/jmrs.533

CPD Questions https://doi.org/10.1002/jmrs.547

**Table:** 

**Questions**	**Answers**
1	B
2	D
3	D
4	C
5	A

Najem F, Prosser S, Harris J, Beldham‐Collins R, Cross S, West K. Radiation therapist‐led telephone follow‐up: identifying patients who require post treatment care. *J Med Radiat Sci*. 2021 Dec; 68(4): 389–395. https://doi.org/10.1002/jmrs.521

CPD Questions https://doi.org/10.1002/jmrs.528

**Table:** 

**Questions**	**Answers**
1	D
2	B
3	D
4	C
5	C

### Volume 69, Issue 1 March 2022

Sapkaroski D, Mundy M, Dimmock MR. Immersive virtual reality simulated learning environment versus role‐play for empathic clinical communication training. *J Med Radiat Sci*. 2022 Mar; 69(1): 56–65. https://doi.org/10.1002/jmrs.555

CPD Questions https://doi.org/10.1002/jmrs.571

**Table:** 

**Questions**	**Answers**
1	D
2	B
3	B
4	D
5	C

Richardson H, Kumar M, Tieu MT, Parker J, Dowling JA, Arm J, Best L, Greer PB, Clapham M, Oldmeadow C, O'Connor L, Wratten C. Assessing the impact of magnetic resonance treatment simulation (MRSIM) on target volume delineation and dose to organs at risk for oropharyngeal radiotherapy. *J Med Radiat Sci*. 2022 Mar; 69(1): 66–74. https://doi.org/10.1002/jmrs.552

CPD Questions https://doi.org/10.1002/jmrs.567

**Table:** 

**Questions**	**Answers**
1	C
2	C
3	B
4	A
5	D

### Volume 69, Issue 2 June 2022

Yeung P, Pinson JA, Badawy MK, Lawson M, Leong C. COVID‐19 pandemic and the effect of increased utilisation of mobile X‐ray examinations on radiation dose to radiographers. *J Med Radiat Sci*. 2022 Jun; 69(2): 147–155. https://doi.org/10.1002/jmrs.570

CPD Questions https://doi.org/10.1002/jmrs.575

**Table:** 

**Questions**	**Answers**
1	C
2	C
3	B
4	A
5	A

Crowe S, Luscombe J, Maxwell S, Simpson‐Page E, Poroa T, Wilks R, Li W, Cleland S, Chan P, Lin C, Kairn T. Evaluation of optical 3D scanning system for radiotherapy use. *J Med Radiat Sci*. 2022 Jun; 69(2): 218–226. https://doi.org/10.1002/jmrs.562

CPD Questions https://doi.org/10.1002/jmrs.568

**Table:** 

**Questions**	**Answers**
1	B
2	C
3	D
4	D
5	B

### Volume 69, Issue 3 September 2022

Williams I, Baird M, Schneider M. Comparison between radiographers with sonography education working in remote Australia and radiologists' interpretation of ultrasound examinations. *J Med Radiat Sci*. 2022 Sep; 69(3): 293–298. https://doi.org/10.1002/jmrs.576

CPD Questions https://doi.org/10.1002/jmrs.596

**Table:** 

**Questions**	**Answers**
1	D
2	C
3	A
4	B
5	A

Knibbs V, Manley S. Being away from home for cancer treatment: A qualitative study of patient experience and supportive care needs during radiation therapy. *J Med Radiat Sci*. 2022 Sep; 69(3): 336–347. https://doi.org/10.1002/jmrs.578

CPD Questions https://doi.org/10.1002/jmrs.592

**Table:** 

**Questions**	**Answers**
1	A
2	C
3	B
4	A
5	D

